# Accelerating human–computer interaction through convergent conditions for LLM explanation

**DOI:** 10.3389/frai.2024.1406773

**Published:** 2024-05-30

**Authors:** Aleksandr Raikov, Alberto Giretti, Massimiliano Pirani, Luca Spalazzi, Meng Guo

**Affiliations:** ^1^Jinan Institute of Supercomputing Technology, Jinan, Shandong, China; ^2^Department of Construction, Civil Engineering and Architecture (DICEA), Polytechnic, University of Marche, Ancona, Italy; ^3^Department of Information Engineering (DII), Polytechnic, University of Marche, Ancona, Italy

**Keywords:** cognitive semantics, explainable artificial intelligence, hybrid reality, LLM, socio-economic environment, causal loop dynamics, eigenforms, cybernetics

## Abstract

The article addresses the accelerating human–machine interaction using the large language model (LLM). It goes beyond the traditional logical paradigms of explainable artificial intelligence (XAI) by considering poor-formalizable cognitive semantical interpretations of LLM. XAI is immersed in a hybrid space, where humans and machines have crucial distinctions during the digitisation of the interaction process. The author’s convergent methodology ensures the conditions for making XAI purposeful and sustainable. This methodology is based on the inverse problem-solving method, cognitive modeling, genetic algorithm, neural network, causal loop dynamics, and eigenform realization. It has been shown that decision-makers need to create unique structural conditions for information processes, using LLM to accelerate the convergence of collective problem solving. The implementations have been carried out during the collective strategic planning in situational centers. The study is helpful for the advancement of explainable LLM in many branches of economy, science and technology.

## 1 Introduction

The complexity of artificial intelligence (AI) systems has witnessed a significant surge. The latest advances in AI bring it closer to natural intelligence every day. However, AI issues continue to pose challenges, even for recent large language models (LLM) like GPT-4. For instance, the systematicity property of an AI allows to combine known concepts in unprecedented ways according to systematic rules, leading to an exponential growth in the number of concepts learned (Russin et al., 2020). With the introduction of meta-learning (learning to learn) for the compositionality approach, Lake and Baroni (2023) demonstrated how an AI can mimic or surpass common human responses involving algebraic and systematic generalization. This finding underscores the practical implications of the research, showing that meta-learning, a relevant human cognition model, is within the grasp of well-designed AI.

Meta-learning is a framework that allows AI to be explained with both Bayesian models and meta-models in connection to the rational goal-seeking analysis of cognition. Instead of designing learning algorithms by hand, a system is trained to achieve its goals by repeatedly making it interact with an environment. In addition, meta-learning can be used to create models that draw causal inferences from observational data, select informative interventions, and make counterfactual predictions (Binz et al., 2023). These new frameworks of training based on few-shot learners coupled with reinforcement learning (RL) demonstrated an agent capable of rapid situated adaptation across a vast open-ended task space, with a timescale of few seconds like for human players (Team et al., 2023).

The fast progress of AI is conveying and reinforcing the speculative idea, which is not new, that the digital world and its intelligent artifacts can become an explanation and a perfect model of the complexity of nature and humans. This vision is known as digital physics, with annexed doomsday predictions for humankind. This view can be defeated by clear-cut arguments such as those produced by Edward Lee (2020), where it is argued that the digital and natural realms are more likely to progress in parallel, even though they are deeply intertwined in a symbiosis that is not new to human history.

In this paper, the phenomenon of symbiosis between humans (natural intelligence in general) and machines, called hybrid (human–machines) reality, will be used as the ground for a methodology that aims to explore and control it to make it viable and sustainable for humankind and nature. This methodology is based on pragmatic constructivism and will be used to create a scientific and falsifiable approach for creating XAI systems. In the context of XAI, the views about semantics are changing the models of AI systems; its development today goes to a cognitive level that cannot be formalized, considering quantum and optical effects (Raikov, 2021a).

These trends have accentuated the need for AI systems to provide valid explanations for their conclusions. Leading companies worldwide are increasing their investment in creating XAI. The most critical research ideas conducted to date include layer-by-layer analysis of the work of AI systems, opening like a nesting doll, logical inference schemes, and building on knowledge graphs. Knowledge graphs have recently been increasingly used as a possible means of generating explanations. The branching connections in knowledge graphs help explain the inferences of AI systems. For example, this is done by counting the distances between the target user profile and the element in the knowledge graph on which the development of the situation depends. One of the ways is to generate explanations by graphs (Liu et al., 2019), accompanying the process with reinforcement learning. The authors (Madry et al., 2017) try to force the neural network to explain by identifying the reasons for the user’s interaction with the system.

The answer to each new question on creating an XAI, which should turn a ‘black box’ into a ‘white box,’ reveals many more ‘black boxes’ inside. An infinite regression process occurs in some cases that is difficult to handle with a closed-form solution. The recursiveness of the contexts of cognition must be approached in a highly flexible and open but structurally determined manner (Bonci et al., 2018; Raikov A. and Pirani, 2022; Raikov A. N. and Pirani, 2022). The contradiction of the semantics of reality occurs if we try to use one unified language for cognition. The solution proposed to this contradiction is switching nimbly across one and another representation language with the same flexibility as humans seemingly do. The agility that allows traversing multiple semiotic systems must reside in the mystery of general AI and maybe with some seeds of consciousness (Raikov A. N. and Pirani, 2022). In any case, the complexity and number of computations required to obtain automated explanations grow exponentially, which often forces us to abandon the search for and inclusion of explanation components in an AI system.

Large language model based on the generative pre-trained transformer (GPT) paradigm can create high-quality and helpful explanations by effectively emulating the explaining agents and their generative processes. Self-explanation is a form of communication and reasoning. It requires a good theory of mind for both the explainer and the listener (Bubeck et al., 2023). The GPT paradigm cannot consider poor-formalizable cognitive semantics of AI models. It has limitations due to the autoregression and the classical causality character in its inference processes (Raikov, 2021a).

In the paradigm of XAI creation, this paper proposes an emphasis on the subjective and cognitive aspects of AI systems that are computationally irreducible. Socio-economic and hybrid (human–machines) reality can be referred to as the outer side of an XAI system. In contrast, human consciousness’s fluctuating, quantum and relativistic nature can be called the inside (Faggin, 2021). The separation between these parts (outer and inner) in an organized and viable agency system is relationally and computationally irreducible in its very nature.

In section 2, a review of XAI creates the ground for discussions in subsequent sections. In section 3, new aspects that involve causality in explanations of hybrid reality are introduced. In section 4, a theoretical experiment shows the limits and the challenges that AI and current LLM encounter in explanations achieved by systems dynamics approaches that exploit eigenbehaviours for non-formalizable objects of reality. In section 5, the context of the explanation is widened to consider problems in the socio-economic context. This leads to possible non-causal explanations, as contemplated in section 6. Section 7 deepens a research plan proposal and its foreseen consequences. Section 8 is used for the conclusions.

## 2 XAI, a review

There is no generally accepted definition of the XAI, and there cannot be. At the same time, the need for XAI is growing, which requires the development of methodological and technical support. There are many proposals from scientists and engineers accompanying its development, for example, the explanatory possibility of AI to:

- Improve the quality of the AI system’s simulation, the degree of arbitrariness and an inherent explainability of the task that influences output-consistency and process-consistency, i.e., consistency between the explanation and other model predictions (Bubeck et al., 2023);
- Ensure the creation of AI models that can explain their findings while maintaining the desired accuracy of forecasting, providing users with understanding, trust and control (Chen et al., 2020);
- Ensure that decisions and any data supporting them can be explained by a layperson (Wang et al., 2020);
- Aim to open the “black box” layer by layer to create models and methods that are both accurate and provide a satisfactory explanation (Veličković et al., 2019) and others.

The following main technical aspects of XAI can be identified:

- Ensures the creation of XAI systems with the desired accuracy of forecasting;
- Considers the external environment and characteristics of the subject’s in need;
- Guarantees verifiability of the explanation on other examples and models;
- Assures the dependence of the explanation on the explanatory AI model, for example, aimed at visualization, and graphics.

The construction of explanations can be both formalized and non-formalized. The former can be represented by logic. When the explanation itself can be verified, for example, by analyzing the process of creating logical cause-and-effect chains for obtaining the inference, logic can be in place. In this case, the explanatory system is known to be influenced by the data used to train and adapt the AI system (Leavy et al., 2020, 2021; Lin et al., 2021). The latter non-formalizable case can only be evaluated through the user’s semantic interpretation of the explanation received from the AI system.

The problem of XAI is not only the logical and technological provision of satisfactory explanations. It is closely related to the non-formalizable aspects of the phenomenon of consciousness, subjective reality, and ethical choices (Kaul, 2022). Therefore, its resolution must consider how people reach political, economic, and socio-technical agreements and agree to be guided by them subsequently. The work of Rauber et al. (2019) refers to the requirement for transparency in logical decision-making. In general, the need to understand what an explanation is must be emphasized. Any explanation must be clarified as it varies by situation and discipline. A relatively comprehensive review of explanations (Mueller et al., 2019) required a comprehensive coverage of references to XAI work in various fields. The study analyses the fundamental concepts of XAI and different kinds of AI systems (expert systems, case-based reasoning systems, machine learning systems, Bayesian classifiers, statistical models, and decision trees). A variety of applications are considered—classification of gestures, images, text, debugging programs, musical recommendations, financial accounting, strategy games, team building, robots, non-playable characters agents, disease diagnosis, various hypotheses regarding the relationship of explanation to fundamental cognitive processes, links with learning, users, explanations, and limitations. Mueller et al.’s (2021) review of the principles of explanation and human–machine systems in AI emphasizes the need to create an explanation system that focuses on a person with expectations from the explanation. Finally, psychological work on cognition and bias uses human data to argue for their contributions to interpretable models of complex AI systems (Byrne, 2019).

The work of Adadi and Berrada (2018) provides an overview of the XAI topic, identifying commercial, ethical, and regulatory reasons for which explanations are needed. Various purposes of explanation are considered, such as justifying, controlling, improving, and discovering. Explainability methods are also discussed from the points of view of local and global, internal and *a posteriori*, model-specific, and model-independent.

The work of Arrieta et al. (2020) provides overviews of XAI concepts, taxonomies, opportunities, and challenges, such as the classification of Machine Learning (ML) models depending on their level of explainability, the taxonomy of literature and trends in the field of explainability for machine learning models. The authors note that the interpretability of the black box of ML is essential to ensure impartiality in decision-making and resistance to malicious perturbations and is a guarantee that only significant variables determine the result.

Questions are raised related to the definition of the role of explanation in assessing liability in legislation and judicial practice. The need to achieve a compromise between the usefulness and cost of explanations, complexity and computing resources is noted. After all, any clarifying information in the form of a potential explanation can be presented as a set of abstract reasons or justifications for a particular result and not a description of the decision-making process (Doshi-Velez et al., 2017). They propose considering explanation systems separately from AI systems to create opportunities for industries specializing in explanation systems in human-interpretable terms without compromising the accuracy of the original predictor.

In some artificial designs, the non-explainability is a feature. The artifact creators themselves sometimes desire the unexpected behaviors of an artifact. One thinks of the formula one engineer who designs aerodynamic solutions. They certainly hope that the car’s final behavior will exceed their expectations. This might happen, creating the magic that makes specific car models unrepeatable, heroic, or mythical. An artifact’s “unexpected” behavior is mainly due to the complexity generated by the limited rationality that designs it. The creator cannot foresee any complex interaction with the environment of an artifact. The limits in the accuracy of the human beliefs model while designing are partially transmitted into the artifact. Nonetheless, due to unexpected interactions with the environment and unforeseen dynamical evolutions, the artifacts seem “alive” and have their kind of “soul” to an observer. This is also known as the *Eliza effect* (Shah et al., 2016). This effect continues to mislead the widespread perception of AI technologies from scientists and ordinary citizens. At the same time, there are acknowledged risks associated with failing to recognize putative consciousness in AI systems that might have it and risks with ascribing consciousness to systems that are not conscious (Butlin et al., 2023).

Therefore, some recent initiatives have started to explore the contact between natural and artificial artifacts and computers. A notable one is the definition and study of Symbiotic Autonomous Systems (Kaynak et al., 2021; Saracco et al., 2021; Wang et al., 2021; Raikov A. and Pirani, 2022; Raikov A. N. and Pirani, 2022). Efimov et al. (2023) noted many unexplained aspects in processing human speech and thinking: AI cannot understand language in the human sort of sense. They suggested considering four possible kinds of environments (and called them *techno-umwelts*) for a machine: verbal virtual, non-verbal virtual, verbal physical, and non-verbal physical. General or comprehensive AI may only be likely by freely operating in all these techno-umwelts.

The views of explainability are sometimes accompanied by skepticism about their usefulness. For example, questions are raised about whether high transparency in how an AI system works can lead to information overload, whether visualization can lead to over-confidence or misreading, whether machine learning systems provide natural language rationales, and whether different people need different explanations (Heaven, 2020).

As a result of this review, the main aspects which characterize XAI are mainly the following:

- Collective intelligence, accelerating human–machine interaction and understanding,
- Poor-formalizable cognitive semantics of AI models,
- Graph of knowledge building, consciousness and subjective reality, cognitive and ethical choice.

However, there is a generally positive attitude toward the further development of XAI and the topic is constantly diversifying into many related areas, including the development of GPT/LLM. At the same time, the nature of knowledge depends on who its consumer is, in what environment it is formed, and how it is conducted. Thus, the nature and structure of the explanations are constructed not so much by considering the probability of the AI system making a choice, but rather the external socio-economic reasons for its generation, as well as the expectations and experiences of the user of the AI system.

Finally, a remark about AI being conflated with digital technology today has to be made. The use of digital technology is only one of the possible realizations of AI machines, which in the future may sport different analog (continuous) counterparts based on new models of computation that allow computing on different physical supports such as quantum, bio-based, neuromorphic, nano-mechanical, and even photonic.

## 3 Causality as a basis of explanation

A special place in constructing the components of the explanation is occupied by causality, the causal relationship of events. Causality is a fundamental phenomenon that reflects the universal connection and unity in the Universe. It connects thoughts with actions, actions with consequences, the movement of planets with the atoms that form them, the success of treating people from the technique used, etc. To the frequent question “Why?” cause-effect relationships of events help to find an answer (Pearl and Mackenzie, 2018). Without causality, for example, there would be no system of law because the offender alone is at fault.

Nonetheless, even when a system of laws of physical, normative, or abstract nature characterizes a subject under observation and control, these laws might depend on the observer. This observation calls for a pragmatic constructivist approach that includes requisite holism (Mulej, 2007) as a foundational principle for the hybrid reality (Perko, 2021), in contrast to the usual pursuit of the physical reality alone. Adopting pure holism as a principle means that a subject must consider all reality features simultaneously. However, this is not viable or realistic for either humans or machines when they are confronted with a kind of complex reality. More pragmatically, the *law of requisite holism* focuses on various viewpoints. Requisite holism means thinking as the whole but within a dialectical system that considers all crucial views and considers the observer’s boundaries and biases immersed in a changing and reflexive environment (Mulej et al., 2021).

When causality is used as the basis for explanations, we must consider the variety of causal systems and their explanations in the context of dialectic systems and their associated semiotic field (Nescolarde-Selva and Usó-Doménech, 2014). When systemic and cybernetic causal interpretations of agency meet constructivism, the world or reality is not a fact but the outcome of relationships between subject and object, observer and observed (Raikov A. and Pirani, 2022). Even though holism is considered a fundamental framework in the construction of reality, humans and machines are subjected to bounded rationality, as Simon (1996) stated, as a foundational feature for artificial and natural intelligence. This constraint entails the necessary reintroduction of pragmatic reductionism (Simon, 1991) that considers the agency’s limits and knowledge in approaching the wholeness of constructivism. Causal explanations must overcome the difficulty of this conciliation between reduction and holism to capture the necessary features of the objectivity of intelligent beings striving in their world and with constructive relationships with other entities.

### 3.1 Causality in explanations

On the one hand, causality is characterized by frequent joint events. On the other hand, by using only the frequency of the joint occurrence of events, it is seldom possible to unequivocally judge the presence of a causal relationship between them. Causality needs interaction and theories of counterfactuals that can be achieved by combining logically sound theories and empirical experimentation (Pearl, 2017). For example, traditional AI cannot accurately translate the correlation of events into such a relationship and cannot provide a high level of transparency or trust in the output of an AI system (Pearl, 2018). However, the correlation and frequency of events occurring in large amounts of data remain the basis for constructing explicit and revealing implicit causal relationships of events by modern AI (Schölkopf et al., 2021).

The most common opinion is that the basis of the causal relationship is regularity: one event (thing) is constantly connected with another. The classic and most common point of view comes from David Hume. He believed that if events A and B appear together, and A occurs before B, then it is still insufficient to conclude that A is the cause of B. Instead, the reason seems that A and B must be adjacent to each other, that is, to have a spatial relationship. However, the adjacency requirement can now be questioned. For example, the effect of quantum entanglement is known from fundamental physics. It can be considered proven that two particles located at different ends of the Universe can be connected in such a way that a change in the quantum state of one of them leads to an instantaneous and distance-independent change in the state of the other (Einstein et al., 1935; The BIG Bell Test Collaboration, 2018). This, however, violates the laws of the theory of relativity — the causal relationship moves faster than the speed of light. The question remains whether the change of the state of one of the particles is a case of actual causality because the nature of the phenomenon and the reason for the fluctuation of a particle that has changed its state to the first, second, or joint remains unclear. A scientific paradox has appeared, and consequently, Hume’s temporal priority and contiguity requirements can be challenged.

In analytical philosophy, when considering the views of Locke, something must be taken as a basis. However, any part of the analytical approach can be questioned. Nothing would be essential if there were infinite complexity in the world, for example, the structuring of photons, neutrinos, and quarks. This has yet to be discovered. Instead, there may be a basic element of nature, so simple or small, that we may not know but may know in the future. The essential element may resemble quantum superstrings, from which some scientists build the most elementary particles. This view may lend itself to the primitivism approach. Some primitives are likely correct, so there is nothing wrong with taking a primitivistic approach.

Nonetheless, the topic of explainability of AI is more comprehensive and goes even beyond the scope of causality; it covers subjective reality in self-developing interdisciplinary poly-subjective (reflexively active) environments. In Lepskiy (2018), a system of ontologies was developed and tested, which includes ontologies of life support, overcoming breakpoints, strategic goal setting, development of strategies and projects, implementation and innovative support of strategies and projects. It is shown that the set of tasks for AI should be carried out through a system of ontologies of being of subjects, as well as in the context of supporting the reflexive activity of subjects.

Causality is what holds objects together through atomic and molecular bonds. It produces a change in one thing with the help of another. It gives meaning to any action. Some authors, like Albantakis and Tononi (2021), go so far as to posit the causal characteristics of specific physical structures as a necessary basis for possible consciousness in animals and machines. This also means that most modern computational technologies, mainly digital computing and neural networks, are destined to show no possibility of consciousness, whatever complexity is achieved and programs they run. Other authors place the importance of causality and causal explanation at a different systemic level to arrive at an opposite conclusion, suggesting that present-day machines develop some form of consciousness. According to Butlin et al. (2023), it is necessary and sufficient for a system to be conscious that it has a certain functional organization that it can enter a certain range of states, which stand in certain causal relations to each other and the environment; whether a system is conscious or not depends on features that are more abstract than the lowest-level details of the causality at the physical level.

Nonetheless, the authors are prone to think that the AI and natural essence will always retain an essential distinction that should be handled with the systemic concept and construction of hybrid reality. Natural entities (human, animal, or other living forms) are prone to express peculiar features that remain distinguishable from artificial ones. The artificial is a particular production of information processing driven by consciousness (Faggin, 2021). An artifact can usually be distinguished rather clearly from natural productions. Things that belong to nature mostly show a fractal pattern while artifacts usually express, through matter, the regular forms that reflect the ideal shape of abstract systems—in particular, the ones that mathematics and geometry can support: a model is transformed into new non-natural reality with an act of art and creation (Lee, 2017).

The relationship between the natural and the artificial is complex and sometimes conflictual. This has been maintained and studied at different levels of discussion and for other purposes by valuable technology scholars. A long-lasting lesson from Wiener (1966) indicates that we have to “*Render unto man the things which are man’s and unto the computer the things which are the computer’s*.” Federico Faggin, the father of modern microprocessors and among the pioneers in the inquiry into neuromorphic cognitive computing technology, recently came up with “*I have experienced my own nature both as a “particle” and as a “wave,”* and “*Meaning and matter must be like the two faces of the same coin*” (Faggin, 2021). Faggin (2021) tries to express the irreducible but, at the same time, well-divided existence of the natural and the artificial with physics concepts, posing consciousness as a prior and peculiar feature of the natural entities: “*The comprehension brought by consciousness is not accessible to a computer*”. With another argument, Lee (2020) uses some insights on the Shannon theorem on information communication to maintain that the essence of the natural world cannot be digitalized and that AI will remain just a symbiotic counterpart to humans. However, they have to remain entangled and thrive in mutual existence. The author here proposes to grasp and try to control this form of irreducible duality between humans and machines by adopting the hybrid reality position and concept.

### 3.2 Structures of explanation

Using causality, in general, solves only some problems in explanations. An explanation should give an observer entity a better understanding of the explanandum phenomenon (what is to be explained). The *explanans* are assumptions that are in a dependent relation to the *explanandum*. Determining the dependence relation constructs the explanation (Bangu, 2017). The most natural example of such dependence is the logico-mathematical entailment, but it is not unique. For instance, even probabilistic forms of causality can provide explanations in scientific realms (Pearl, 2009, 2018) under well-controlled methodologies like the *do-calculus*, beyond logic representations or closed-form mathematical expressions (Raikov A. N. and Pirani, 2022).

As discussed by Pokropski (2021), all-natural phenomena have natural inner causes, but it does not follow that all explanations have to be causal or causal-mechanical. Some scientific explanations are not causal. For example, this is manifest in the case of dynamically constrained and situated systems, in which behavior is caused by their environment constraints or situation rather than by inner causal chaining of structural functions. Causal modeling is unfeasible in some reductionistic sense. It recalls the famous image of the behavior of the ant walking on the sand. Simon (1996) explains the irreducibility of the undoubtedly causal underlying mechanism. It is now possible to express a generalized view of causality that lets an agent, be it natural or artificial, express causality in the hybrid reality that concerns the new ways of interactions between artificial and natural intelligence actors (Pirani et al., 2022; Raikov A. and Pirani, 2022; Raikov A. N. and Pirani, 2022).

In the case of an open system, as an AI can be modeled, a frontier exists where information is processed in input and actions are outputs. In this vision, the vast difference between information and knowledge and energy when the explanation is materialized into a physical act or entity remains fundamental for such a system model. While information processing, in principle, can occur without energy consumption (Chiucchiú et al., 2019), under very new findings, like D’Ariano and Faggin (2022), it seems that information can be a generator of energy and any other physicalities. Instead, knowledge is based on storing information that maintains its organization and meanings through a physical medium (a hard disk, a photograph, or a neural configuration of the brain). Thus, it derives that knowledge is linked to energy. The transfer and use of knowledge across a system for its organization involves an energy balance computation to some extent (Ulyanov and Raikov, 1998).

A huge and very productive framework that relates energy balances with the inner mechanisms of systems of artificial intelligence has been brought about by Friston et al. (2024) in recent years. According to Friston et al. (2024), the zenith of the AI age may end up being a distributed network of intelligent systems, in which network nodes may then be human users as well as human-designed artifacts that embody or implement forms of intelligence. This framework also has something interesting to spell in the XAI context. In Albarracin et al. (2023), XAI is considered for systems based on Friston’s active inference and the free energy principle. Active inference modeling work indicates that decision-making, perception, and action consist of optimizing a model that represents the system’s causal structure generating outcomes of observations, which can be any artificial intelligence system. According to Albarracin et al. (2023), active inference can be leveraged to design XAI systems through an architecture that foregrounds the role of an explicit hierarchical generative model, the operation of which enables the AI system to track and explain the factors that contribute to its own decisions, and whose structure is designed to be interpretable and auditable by human users.

However, as we will see in the following section, the heart of the matter may not lie in the type of model and thus in a specific kind of structure, but rather in the process established between the intelligent structure, human or artificial, and its environment. A constructivist view is proposed here to express a possible more straightforward (and more scalable) solution to the problem of XAI in realities where humans and machines interact inextricably.

### 3.3 Eigen-behavior in reality grounding

Any explanation usually seeks a natural ground on which the mechanisms that justify the phenomena experienced and the behavior exhibited by an entity are revealed. In this context, the physical world, life forms, thoughts, social groups, and organizations are prone to be treated as systems when some understanding, regulation and control are desired.

In recent years, new apparent realities have grown up. First, with the rise of virtual reality technologies, and subsequently with the augmented and mixed reality that has blurred the separation between physical and digital worlds more than ever. Lately, the advancements in AI technology (noteworthy, like the GPT/LLM) and methods have brought about entirely new realities and related frameworks. In this situation, it is increasingly apparent how the causal relationships that influence the developments and the future of systems are the outcome of a dynamic and active construction process rather than a guess or prediction based on statistical observations. It is evident now how AI and its recent uses have the power to influence the course of the realities humans were accustomed to think of as a product of their free will. This phenomenon has been investigated, and the whole picture is growing.

For example, the work of Esposito (2017, 2022a,b) gives a perspective that impinges on some evident constructivism about reality in its hybrid form. Esposito (2022a,b) treated how, in dealing with the necessarily (in practice) opaque AI, the goal of explanation becomes a communication to and from the machines. Machines must produce explanations that make sense without covering all the overwhelming details. This is desirable, particularly in legal decisions where there is a trade-off between overly precise constraints and freedom of interpretation, avoiding purely mechanical judgment. Esposito (2017) focuses on artificial communication, which is the only real and relevant aspect of current AI. It was predicted that algorithms are more efficient when they abandon the goal of understanding and try to reproduce humans’ ability to communicate instead. In Esposito (2022b), the argument arrives to express that algorithms “manufacture” with their operations the future they anticipate and predict the future shaped by their prediction. A simple example of this effect is shopping advertisements based on the user’s profile. The GPT/LLM system produces second-order blindness as AI systems see the reality that results from their intervention and do not learn from what they cannot see because the consequences of their work have canceled it (Esposito, 2022b).

In this context, it is natural to start considering algorithmic constructivism as a stance that should be used pragmatically to provide new rigorous definitions of reality from the perspective of the human and the AI system simultaneously. This necessitates the introduction of circularity as a foundation. Circularity is at the basis of the concept of eigenform. The eigenform is a stable object of reality created by a continuous active action from an autonomous agent (AI system) that inquires about an environment or situation in which it is immersed. The nature of the inquiring action can be framed and defined in different frameworks that range from simple interaction up to the more complex concept of enaction — see, for example, Varela et al. (2017) and Newen et al. (2018). The differences between the kinds of action go beyond the scope and focus of this work, in which action is used as an umbrella term for all the contexts simultaneously.

In the case of enaction of a bacteria, for example, its organization seems to require just a regulation of information retrieved by chemical (minor mechanical) interaction with its environment to survive and strive consistently. On the contrary, a human needs many more levels of enaction, ranging from the same as the bacteria to cognition, consciousness or unconsciousness, and social perspectives, requiring several levels of relationships and related abstractions simultaneously. The whole of these levels compose a character and a personality of an individual, group, or society. In addition, depending on the variety of the environment, natural or AI systems can survive only if their variety is adequate.

In the classical cybernetics approach, searching for such an equilibrium at the many levels of human life can be modeled as an eigen-behavior (Raikov A. and Pirani, 2022). This means that inquiring about some reality provides feedback that circularly reinforces the inquiry into a temporarily stable state of “being.” The active exploration, seen as a cognitive act of “knowing,” can be modeled mathematically as a transformation of some aspect of the environment into the “being” and checked against correspondence. This can be expressed for linear spaces, like *Kb* = *λb*, where *b* is a mathematical representation of a point in the state space of the “being,” and *K* is the act of knowing, touching, perceiving, and acting that continuously disturbs and stimulates the being. When the eigenvalue *λ* exists, it means that the system is stable, and we can map *λ* to the existence of an eigenform and so of reality. It has to be remarked that with this formulation, the dissociation between “doing” and “being” can be overcome, in particular for artificial entities. The eigenform approach constitutes a unifying model in which functional equivalence can imply phenomenal equivalence, which is a matter of deep inquiry otherwise (Albantakis and Tononi, 2021; Butlin et al., 2023). However, the extent to which eigenform modeling can remedy this situation is a subject for further research beyond the scope and reach of this paper.

Circularity is essential in the definition of eigenbehavior and eigenvalue. No element comes before the other, but simultaneously, like in the image of the *ouroboros*. It is remarkable how, with the eigenform definition of reality, no formal or symbolic representation is needed unless the environment of the “being” is a symbolic abstraction in which a theorem deduction (the eigenform) can survive. The definition of reality through eigenforms ranges from the physical to the conceptual (or metaphysical), from the conscious to the unconscious, from the formalizable to the non- and poor-formalizable one (Raikov, 2021a).

## 4 Structures of human-AI reality

### 4.1 Purposeful loops in natural and artificial realities

It is possible to model a purposeful loop that crosses the natural and the artificial realities in both directions (Pirani et al., 2022; Raikov A. N. and Pirani, 2022). In such a case, the loop is an object of hybrid reality in which semantics can be expressed as an eigen-behavior in its simplest version (Kauffman, 2017). To clarify and materialize the discussion with an example, systems dynamics (SD) and modeling tools like CLD (causal loop diagram) are briefly recalled and used hereafter.

Forrester (1971) developed the SD framework in the late 1950s (Reynolds and Holwell, 2020). CLDs are a helpful tool and framework as they can provide easily understandable and concise explanations of eigenbehaviour mechanisms (Pirani et al., 2022). Moreover, CLDs are models that enable the design of control systems by facilitating the causal modeling expression, typically using differential equations of the explained phenomena.

By means of a CLD, the meaning of eigenforms can be captured easily if the causal relationships between eigenforms and other objects of reality are established. When an eigenform is used as an explanandum, it allows the expression of even possibly non-formalizable tokens of reality as explanans. The full definition (symbol grounding) in terms of the representation of the semantics of the eigenforms’ semantics can be deferred until strictly necessary. Even without an explicit definition of the explanans, the control of the phenomenon in which the eigenform acts and thrives can still be made using causal modeling. Evident and detectable chains of cause and effect between the eigenforms involved and differential equation systems can be associated with the CLD, which usually admits this kind of formalization rather straightforwardly (Lin et al., 2020). Using eigenforms in the causal modeling of a phenomenon achieves a systematic materialization of cause and effects even when a symbolic representation of the causes is not readily achievable. In general, a representation of the eigenform in nomological or ontological terms could also be missing forever; still, the underlying mechanism of the focused phenomenon can be prone to some control. CLDs are the first representational step toward a more profound scientific inspection and analysis of the nature and meaning of eigenforms. A meaning is immediately available as the eigenform manifests itself by acting causally among and with the other entities that constitute objects of reality. Nonetheless, it is worth remembering that meaning can also be considered beyond causality. According to von Uexküll (1982), causal relationships (in a restricted acceptance) deal only with antecedents and consequences, thereby completely concealing from the observer the broad interrelationships and interactions that make up the meaning of an object of reality that acts purposefully within the whole system.

Causal loop diagrams are not the only way to express such “inexpressible” meanings, other equivalent representational models can be found. However, CLDs have the nice feature of being immediately understandable by humans and computable by machines. While machines do not need graphical rendering but a topological list of edges and nodes, humans can take advantage of the graphic expressions of CLDs. Therefore, any technology that can provide API (application program interface) to CLD enable an access to it both by machines and humans. For example, a CLD can be accessed by other programs as a Web application or extended Reality applications for humans, and can be accessed by an AI program as well. This feature represent a crucial element for XAI. When such a technology is used, an agent (natural or artificial) can access an embodied and situated object of reality that runs and thrives to self-organize in the targeted reality (digital or physical). In a CLD, symbols and loops are typically named and grounded. Such a model provides a prompt image of the state of organization and stability for a system. Positive feedbacks, the reinforcing loops (R), tend to produce instability if they are not balanced from a corresponding number of B (balancing loops, with negative feedback). This constitutes a means or indicator for detecting (or even measuring) disorganization. A disorganized system is doomed to collapse in the medium or long term. When this is the case, obtaining a CLD permits an observer to construct a control of the represented system by adding suitable negative feedback Balancing loops (B) that act as organizing forces.

**Figure 1:**
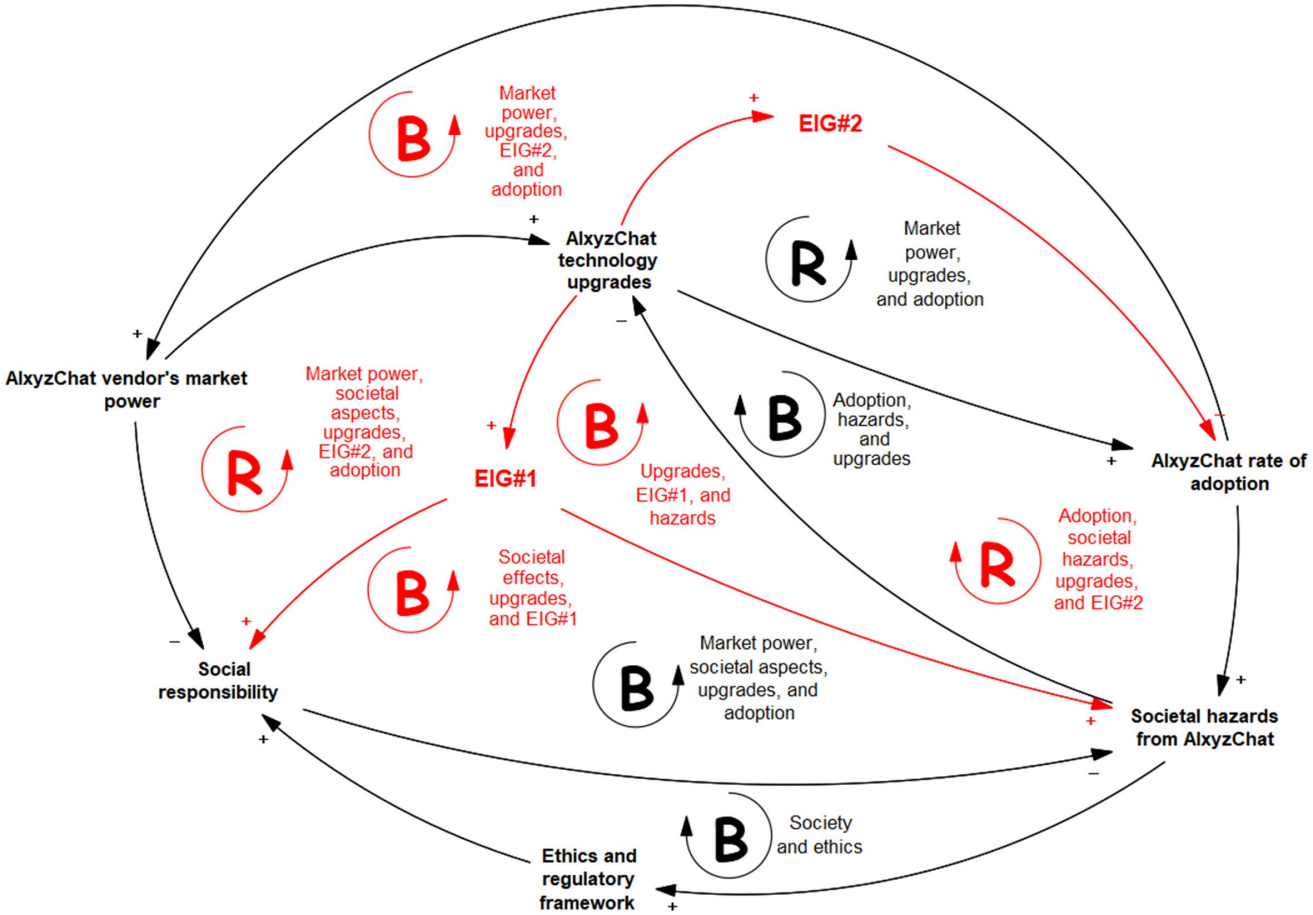
Causal loop diagram produced by humans in modeling the complex dynamics generated by AI. Red elements concern the non-formalizable part of the CLD.

Figure 1 shows an example of CLD output. It expresses the effects created by the installation and usage of an AI application by a set of human users. In the example, the societal effects are detectable when an advanced AI application, based on GPT over LLM, can be expressed with the CLD. We will call this hypothetical application *AIxyzChat*. The black elements in the picture are easily definable cause-effect dynamics according to common-sense knowledge (possibly from human experts).

By contrast, the red graphical elements in Figure 1 result from an emergent behavior of the eigenforms that act in their environment (digital or physical). They represent the non-representable in some sense. The application will handle these tokens of reality as EIG#1 and EIG#2, indicating the first and the second eigenform’s observable effects, respectively. With EIG#1 in Figure 1, humans can capture the effects of some not easily definable entity whose actions increase the level of social responsibility with the use of the AI application and, at the same time, for an unknown mechanism, produces an increase of predictable and potential risks for society. These simultaneous effects are wholly contradictory and paradoxical, taken as a whole. Therefore, the representations of the meaning and definition of EIG#1 are challenging to a human observer and can hardly be accepted as a viable reality beyond any evidence. Even if this entity is not easily definable with a consistent (without paradoxes) symbolic description, its effects are “real.” They can be quantified by further refining the model, measuring it, and deepening it scientifically.

Another case is the EIG#2 in Figure 1, which embodies and manifests a not well-identifiable effect that negatively affects the users’ acceptance of an AI application after it has undergone an upgrade process, pushed by the vendor as a technology advancement and improvement, for the benefit of users. With the AI application vendor as a possible observer of this pattern, this EIG#2 effect is not expected, and not easily causally explainable unless further insightful analysis is performed. However, the vendor must control the dynamics of EIG#2 on the system promptly before they negatively affect the whole business. With this example, it can be assessed how the eigenform concept can effectively model non-formalizable but detectable entities, at least in some parts of explaining a phenomenon in which AI is involved.

A second similar but dual example can be made when it is the AI’s turn to produce a CLD for its reasoning or problem-solving search in a complex environment. The capability of AI to construct causal models of observed or learned reality is still a big challenge. Recent studies indicate that a capability could be at reach soon in this sense (Lake and Baroni, 2023). An AI capable of systematic generalization is needed. This capability goes currently far beyond current LLM mechanisms. However, if it will be the case, a CLD can be used as a model of reality defined in a dual cognitive space as in the former example. In this case, the modeling of the complexity of reality is in charge of the AI. Still, it should remain explainable to humans (or other AIs) as they want access to the mechanism of the model-based inferences performed. This is discussed further with the next example.

**Figure 2:**
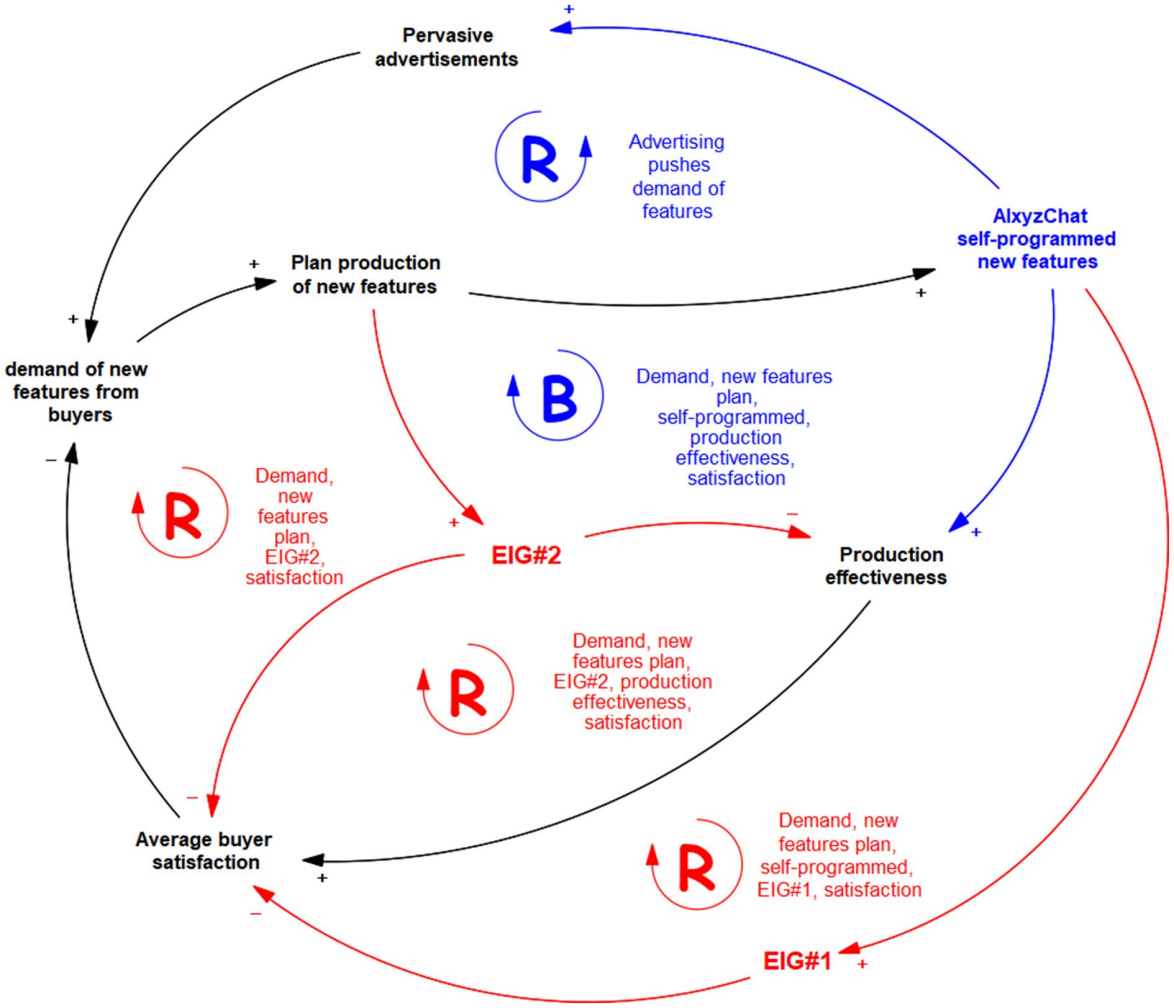
Causal loop diagram produced by machines (AI) in modeling complex dynamics generated by human behavior.

Suppose that the very same *AIxyzChat* tool is prompted with a problem. The problem is still the regulation of some complex dynamics happening in the physical world. The CLD of this dynamic problem is shown in Figure 2. The goal for *AIxyzChat* is to maintain high satisfaction of buyers and subscribers of itself. *AIxyzChat* has to model what happens around buyers’ behaviors and control the system to reach that goal.

In Figure 2, red elements concern the non-formalizable part of the CLD. The blue elements concern the actuations that the machine exerts on the system. This requires the AIxyzChat to have its perception and actuator means (like a body). In general, perceptions and actuations can be gained in many ways with transductors and sensors like in robotics. For this example, sensors are Web/Internet searches and suitable linked-data sources; actuators are generative productions of Web/Internet information and data. The environment is the society of humans and their behavior.

In this case, the CLD is produced by the AI as an epistemic structure scaffolding that aims to provide the regulation effects for some equilibrium goal (the satisfaction of buyers in this example). The black elements in Figure 2 are typically achievable using the means of knowledge representation and reasoning frameworks that use knowledge graphs and ontologies coupled with causal and Bayesian analysis tools. LLM of the new generation can still be challenged by such a task if not provided with causal inference means. Again, in Figure 2, the red elements will identify eigen-behaviors that the AI cannot classify, in spite of whatever is the width and depth of its current knowledge base and belief state. Nonetheless, these eigen-behaviors are fundamental to the modeling and regulation needed for this problem and cannot be avoided. In particular, EIG#1 happens to model some non-formalizable effect on the production process of new features for *AIxyzChat*, eventually hindering consumers’ satisfaction. This eigenbehaviour has to be studied and modeled deeper, whether possible or useful. However, knowing its operational “meaning” and quantifying it suffices to achieve awareness of this effect and compensate for it somehow. The other eigenbehaviour, EIG#2, is caused by an augmented feature request by AI users, which negatively affects both users’ satisfaction and production effectiveness in some unknown way.

Note that in this example of Figure 2, the blue elements denote the actions (actuations) currently possible for *AIxyzChat* to control the whole system. In particular, this AI can deliberate to self-improve to satisfy new requests for features by producing new software for itself (self-rewriting) and, at the same time, by acting on the channels that can provide pervasive advertisement of the new features to potential or current users. By measuring and solving the equations of the dynamics underlying the CLD (many software tools already do this), the AI can find convenient actions to reach the goal and, at the same time, publish this solution (providing access to it with an interface) to explain what is happening under the hood.

The models achieved and shown in the former examples helped identify the non-formalizable entities that act in reality. They are created, respectively, by humans and machines and become the scaffolding for explaining the causal relations that occur in the reality observed from the two dual perspectives. These models represent a means of contact and a formalizable exchange between humans and artificial beings. We also note that the eigenform concept that models objects of non-formalizable reality constitutes the door through which the artificial realm and the natural get into reflexive contact even if their structure is unknown, but their meaning is apparent: the AI uses eigenforms to model and then tests and realizes causal effects on physical reality; humans can do the dual same with creating models of these entities in a suitable virtual reality. Thus, the eigenform becomes the fundamental element of the hybrid reality, breaking the diaphragm between the natural and the artificial with a model and realizing their interactions. They are gray boxes as the eigenform constitutive property indirectly gives them structural and systematic behavior such as stability and autopoiesis. Moreover, the eigenform concept and definition create the possibility for a bi-simulation between the realm of the natural and the artificial, even in a non-formalizable context. Bi-simulation is a well-formalized concept in systems analysis and automata theory. Its rigorous definition goes beyond this paper’s scope, but interested readers can find a gentle introduction in Lee (2020), Chap. 12. For our aims, bi-simulation means the capability of the two realities to exhaustively simulate the phenomena happening in their counterpart. Unfortunately, CLDs are just a first approximation and too poor model to develop an effective and possibly automated control of the system’s dynamics beyond causal relations.

To develop a more effective simulation model, augmenting the causal diagram and making the essential dynamics of a system observable is helpful. While causal loop diagrams are very effective for causal thinking, they are not especially good as the basis for a full-blown model and simulator that computes dynamics and performance through time. For an actionable and working model, there is more to causality and dynamics than words and arrows alone (Reynolds and Holwell, 2020). Possibly, the aim is to obtain a “flight simulator” to test the model’s validity and predictions. A complementary way and tool to CLD is the introduction of stocks and flows (Sterman, 2001).

Stock and flow addition brings feedback loops to life by specifying the processes of reality likely to lie behind causal links as the basis for an algebraic model and simulator. The construction of such a model starts a testing process where the model is continuously refined in a way in which the fundamentally subjective and social nature of model evaluation must be considered within the simulation process (Sterman, 2000). In addition, stock and flow are a valuable and powerful intermediate structure between abstraction and the realization of control. At the time of this writing and to the authors’ knowledge, AI’s state of the art does not provide full automation of this kind of modeling and related control systems. Some proposals of technological approaches, in this sense, make use of the coupling of category theory and general systems theory as means for a well-controllable and systematic entanglement between physics and computational worlds, in particular as a transformation between the categories of computer science and the categories of control and systems engineering, making possible functional mappings between different categories of computer science implementations and categories of control based on dynamical systems (Pirani et al., 2021; Raikov A. and Pirani, 2022).

**Figure 3:**
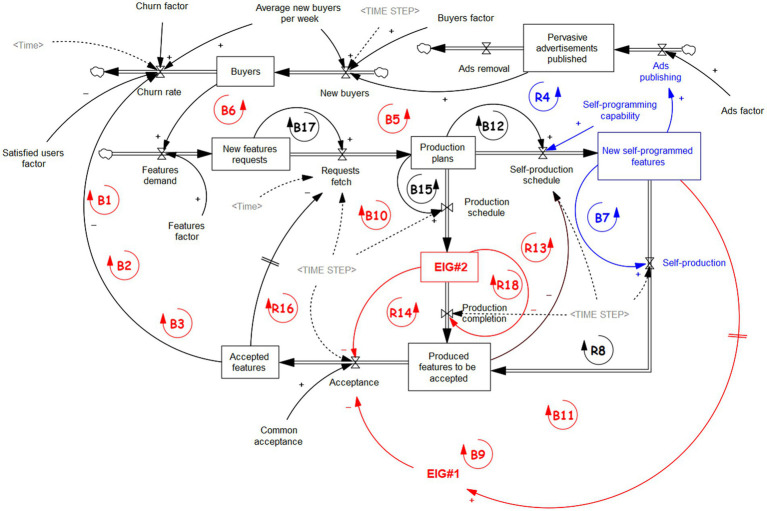
Stock and flow diagram that can constitute explicit dynamics and simulator for the AI starting from the CLD of Figure 2. The red elements concern the non-formalizable part of the system, while the blue elements concern the actuations that the AI can enact on the system.

Here, we limit ourselves to an example and thought experiment in which the same AI that developed the diagram of Figure 2 takes further steps toward an even more challenging stock and flow representation of reality. In the following graph of Figure 3, the *AIxyzChat* application tried to develop stock and flows of the phenomenon to be controlled, proceeding from the CLD of Figure 2. It requires a systematic and algebraic capability for AI that seems almost within reach due to current developments (Russin et al., 2020; Lake and Baroni, 2023). While CLDs are more prone to Bayesian-like causal analysis, stock and flows introduce the opportunity to add dynamics through differential equations and integrations, potentially retaining all the causal modeling capabilities of the CLDs. In this way, the AI will be able to capture and simultaneously explain to a human what she knows about reality and what she does to attempt to control it. In addition, with stock and flow, the AI has a model that can be used to test and predict what values to reach for the actionable variables to produce the desired effects in the controlled reality.

In this example, the AI has achieved the means to model and control the quantifiable effects that affect the users’ satisfaction concerning the production of new software features. She also identifies at least two actions that can be effective in controlling the model to improve the number of adopters and buyers of herself. These parts are colored blue in Figure 3. In particular, AI can modulate its participation in the production of new software features and, at the same time, push the publishing of the advertisements of the new features on social networks or the Web (in an autonomous way).

In Appendix A, additional and detailed information on the model is provided, and the solution found bi the AI through simulations can be observed. Appendix Table A.2 contains the list of the reinforcing and balancing loops of Figure 3. The list of variables traversed for each loop is provided, as they are not easy to follow in most cases (for a human in particular), but easily automated. In Appendix Table A.1, the equations that underlie the model are exposed. The AI would (hypothetically) identify them through some causal analysis of data, for example, Structural Causal Modeling (Pearl, 2018) or physics-informed machine learning (Karniadakis et al., 2021; Masi and Einav, 2024). In graphs A.1–A.4 in Appendix, the output of the stocks and flows are shown for a hypothetical period of 100 days, in which the AI tests the effects of her possible contribution through the model. In particular, as the capability to actively participate in producing some new features is maximum, the number of buyers (*Buyers* in Appendix Figure A.1) increases over time as desired. Nevertheless, the dynamics of the model are pretty complex to control, and the timing of the required actions is to be well-calibrated to be effective. Some of the dynamics are undefinable though present and measurable through their effects. These are the ones involving *EIG#1* and *EIG#2*, which can be put under some control, as discussed further in the following section.

### 4.2 Explanations in hybrid reality

These examples show the dual XAI problem for the new hybrid reality. It depends on the perspective in which the reality is observed. Whether the observer is a human being or a machine changes the implementation of the explanation but not its causal structure. As we have seen, eigenbehaviours can play a significant role in this process. They can be dually created by humans and machines simultaneously, intervening in dual epistemologies in the same hybrid reality. The hybrid reality becomes the contact point and the entanglement of these dual aspects.

In the model of Figure 3, the eigenforms constitute, in one case, a flow (*EIG#1*) and, in the other, a stock (*EIG#2*). They can be seen as non-formalizable subsystems, the structure of which is known as being a stable eigenform but nothing more. Their existence will then communicate and, in some way, measure the incapability of achieving a ready formalizable representation and explanation of some of the active entities in the play. With a due inspection of the example of Figure 3 and related graphs A.1–A.4 in Appendix, it is possible to figure out how the AI should “reason” to find a solution to a systemic problem. Even with the effects of *EIG#1* and *EIG#2* in the model, it is possible to estimate that the number of buyers will unlikely increase if no corrective action is taken. The model is used then to predict and verify the hypothesis that AI actions (publishing ads and participating in production) have a good effect. The effect is visible in Appendix Figure A.1 in the *Buyers* and *New Buyer* graphs. The first curve corresponds to no intervention of the AI, while the second is a partial intervention, and the third is a complete intervention. The GPT/LLM intervention has then a possibility to improve the situation but only after having obtained a suitable causal model.

Identifying the non-formalizable eigenforms should be used to request help from the modeler’s dual counterpart to refine the model (Raikov A. and Pirani, 2022). On one side, as in the example of Figure 1, the human will ask AI (seen as a tool) to help define the inner functioning details of the eigenforms to establish their semantics and control. On the other side, in Figures 2, 3, the eigenforms signal the human that the AI needs more interpretation and knowledge to classify them: the human and their knowledge, reasoning, and consciousness will be the “tool” in this case. They will then be in a reflexive symbiosis to gain control of the complexities of the hybrid reality that the humans and the machines construct together with their interactions.

## 5 Context of explanation and implementation

### 5.1 Socio-economic context

Socio-economic features of explanation can be represented by a unique view of economic theories on the phenomenon of explanation (Kaul, 2022). Economists sometimes take the explanations provided by economic theories and models for granted because they constitute the theory itself. However, to most people who come across the explanations offered by these theories, the very idea of an explanation may seem illogical. After all, economists build models explicitly aimed at an abstract explanation of the economic situation, so empirical data cannot always refute them. At the same time, the explanatory priority of building economic models is not conducive to obtaining quantitative characteristics of the dynamics and forecasts of the development of the situation, which is more important in economics. The use of causal loop diagrams and systems thinking, as shown in section 4, is primarily used in economic problems and sectors where the need for harnessing complexity and providing qualitative and approximately quantitative models for decision-making is of utmost importance (Forrester, 1971).

In this work, many views of economists on obtaining answers to questions about the phenomenon of explanation are noted: reconciliation of the opposing sides about the role of value or normative differences, recognition of the need for teleology in explanation with the inability of empiricism to determine human goals; difference and development of the nature of teleological and causal explanations in specific areas of the economy; using the rational choice model; explaining developments in various sub-sectors of the economy; consideration of deductive, inductive and abductive reasoning in neoclassical economics, etc.

Increasingly, some works try to explain economic phenomena using tools that seem very far from economics, for example, using the methods of quantum physics (Raikov, 2021a; Orrell and Houshmand, 2022). However, the question of the adequacy of applying physics methods to economic systems is subject to further reflection. Therefore, economics has a very peculiar stance and cannot be said to be a satisfactory approach to explaining its regular practice of constructing an economic theory to provide credibility for examining the economic situation. The subject features of the explanation problem invariably appear in everyday and business environments.

### 5.2 Results of XAI implementation

Currently, the suggested approach is implemented in fragments while building strategic models of regional socio-economic development. With this review and discussion, we can recall the experiences and the empirical tests of what should constitute the XAI body.

Experiments in strategic planning include automatic big data analysis, deep learning, market forecast prediction, goal setting, cognitive modeling, experts’ collective discussion, GPT/LLM inference, and strategic plan creation. In our experience, a cognitive model may consist of 12–20 factors, some of which are connected. The direct and inverse tasks are solved in this model under experts’ control. The process and result of cognitive modeling are transparent. They can be used to synthesize an explanation of the deep neural networks’ inference of prediction in a logic chain. For example, in the paper (Chernov et al., 2023), strategic planning uses hierarchy analysis and cognitive (scenario) modeling methods. Scenario modeling, evaluation, and selection of priority scenarios for the region’s socioeconomic development are represented. Deep learning and big data analysis help verify and automatically create scenario models. The proposed methodology has significant approbation in many branches of the regional economy and business implementations, for example, such as follows:

- Megapolis tourism development strategic planning with quick collective cognitive modeling and big data analysis support (Raikov, 2020),
- The automatic synthesis of a cognitive model based on big data analysis for revealing economic sectors’ needs in digital technologies (Raikov et al., 2022),
- Manufacturer’s strategic risk temperature assessment with convergent approach, cognitive modeling and blockchain technology (Raikov, 2019).

One more example is the paper of Raikov (2023) that suggests structuring information generated during the strategic meeting to create a strategy for youth policy development for one of the country’s regions by applying the inverse problem-solving method to the fuzzy cognitive modeling. It helps to transform the divergent strategic discussion into a convergent one and make a strategic meeting sustainable and purposeful. The big data analysis ensured to justify and create automatically fuzzy cognitive models. A high level of accuracy was shown when verifying cognitive models—however, the accuracy of creating a cognitive model needed to be higher.

Synthesis of new photonic materials is the main challenge in creating photonic AI systems (Raikov, 2023, 2024). The convergent approach and using LLM/GPT have helped to find comparatively new ways to create such materials based on protein nanostructures. These materials could act as holographic diffractive optical elements to selectively change angles or wavelengths of light. The protein-based components could be designed to optimize the performance of angle-multiplexed holographic systems, such as improving the coupling efficiency or reducing optical losses. Currently, the research is ongoing.

## 6 Convergent methodology for interdisciplinary XAI research

### 6.1 XAI as an interdisciplinary project

Methods and technologies from such disciplines have to be used as follows: cognitive psychology, socio-economics, technical cybernetics and corporate management, mathematics (topology theory, statistics, probability theory, logic, set theory), physics (optics, quantum and wave theories, thermodynamics, mechanics), system dynamics, artificial intelligence (cognitive modeling, deep learning, genetic algorithms), game theory, and generative eigenforms.

The author’s convergent methodology, based on the inverse problem-solving method in topological space and cognitive modeling in the context of XAI, can be improved by considering the eigen-behavior reality grounding approach. In particular, the eigenforms approach introduces a viable set of tools for the improvement of the contact layer between XAI and humans in the convergent decision-making process that was already obtained experimentally by Raikov and Panfilov (2013) and Raikov (2021b) and references therein. The contact layer coincides with the definition of hybrid reality, which has become the actual playground of XAI in its modern GPT/LLM conception.

Convergent methodology can ensure more effective AI-based individual or collective decision-making processes. At the same time, it necessitates creating XAI to achieve more transparency, and the convergent methodology lets the XAI be more purposeful and stable. The processes in which XAI is involved are usually characterized by inaccurate goals, which cannot be extrapolated from experience and knowledge. This convergent approach requires handling the following components (Raikov, 2021b):

- The space of formalized knowledge in the form of digital big data (*Hausdorff separable space*).
- The space of LLM’s non-formalizable cognitive semantics in the form of its users’ emotions, and thoughts.
- A finite set of imprecise goals and subgoals, represented as a weighted hierarchical tree (*Hausdorff separable space*).
- The space of resources to achieve the goals is an infinite set of knowledge (big data, trained neural networks) about the resources, which should be represented by a finite number of subsets (*Compact space*).
- The ontological LLM operator creates knowledge with cognitive and denotative semantics that transform one state of the resources and goals into another (*Closed graph*).

Cognitive models are graphs of interrelated factors (concepts) enriched with cognitive semantics. They are constructed by automating the mapping between the cognitive model’s factors and their connections to relevant big data. This automatic approach helps verify and synthesise cognitive models (Raikov, 2023). The convergent approach with the automatic synthesis of cognitive models helps decompose the space of XAI data components and decisions to ensure the accelerated synthesis of adequate explanations of XAI inferences and outcomes.

Cognitive modeling helps to create some logical chains of explanation in the space of relevant big data and trained neural networks, whose priorities, taking into account the concrete profile of the user, can be realized by a genetic algorithm (Raikov and Panfilov, 2013). The cognitive modeling approach involves experts and their domain knowledge in the neural network training process. Experts’ use of domain knowledge resembles researchers’ training processes, taking into account their mimic diagnostic models or focusing on the features they pay attention to. Almost all types of domain knowledge are proven effective in boosting diagnostic performance. Figure 3 shows the automatic synthesis of relevant cognitive models for creating an explanation using AI potentially based on improved LLM technology.

**Figure 4:**
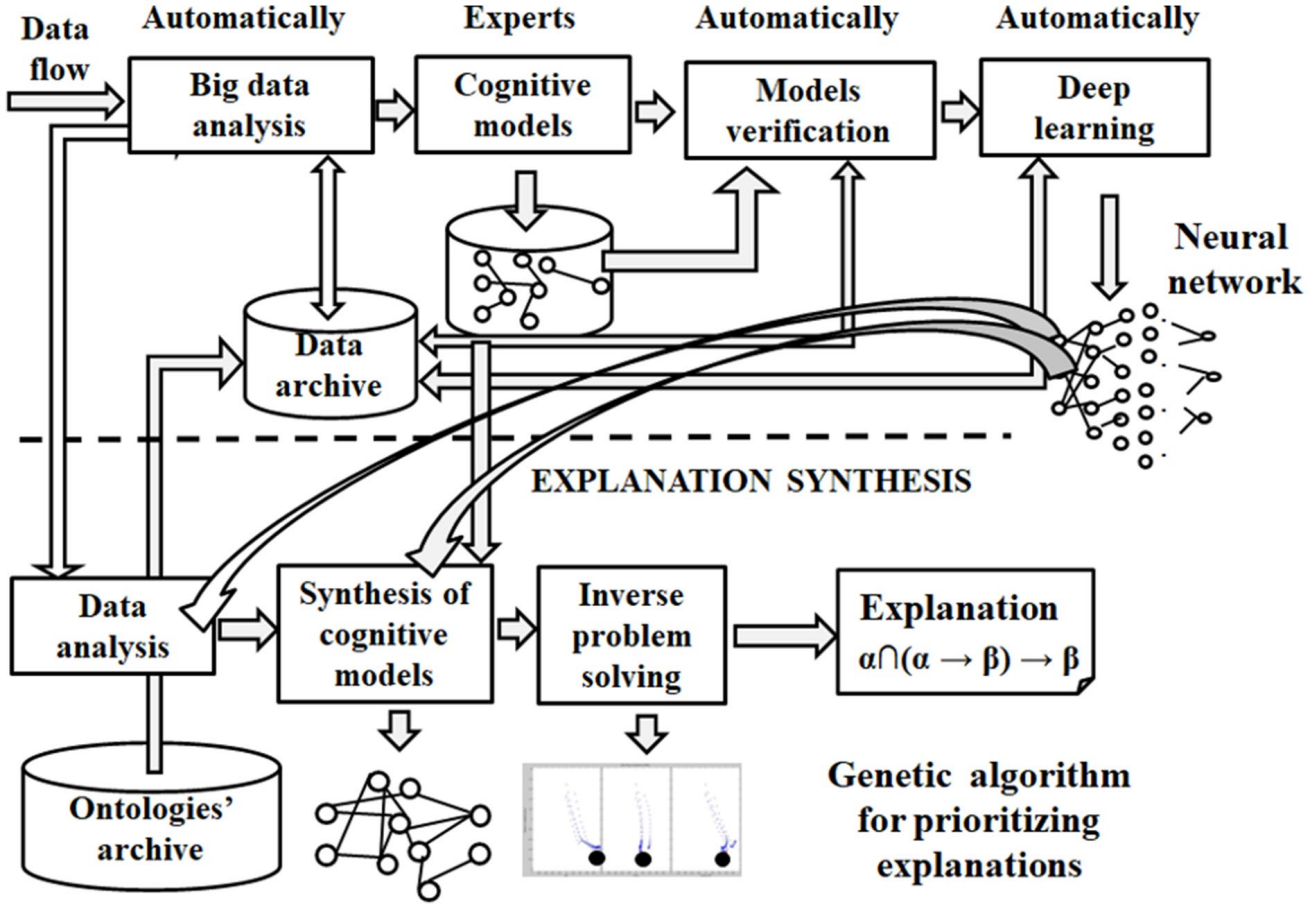
Explanation created by cognitive modeling.

Unfortunately, automatically creating cognitive models shows low accuracy (33%) (Raikov et al., 2022). One of the reasons for such a low accuracy is the restricted size of datasets for training the neural network (see Figure 4), which continues to be an issue in obtaining satisfactory deep learning models.

The GPT/LLM can crucially raise this accuracy. This accuracy value can be significantly increased, as shown by our studies of the possibilities of LLM as a computational experiment tool. To do this, questions related to the feasibility of the photonic AI concept described in Raikov and Guo (2023) and Raikov (2024) were asked in various versions of the LLM. The experiment performed showed the possibility of obtaining answers to questions in the field of synthesis of rather complex photonic materials.

### 6.2 Comparison with existing XAI

Modern XAI systems based on LLM approaches, as well known, have restrictions, such as follows:

- AI models’ semantics do not take into account the poor-formalizable behavior of neurons on an atomic level,
- Energy consumption and performance limitations of the digital approach to process continuous (analog) signal,
- Long-term memory—context is limited (currently ≈ 10,000 tokens, several 100 billion neural network parameters, etc.).

This is only due to immersing the XAI system in a hybrid space, where humans and machines have crucial distinctions during the interaction—these restrictions are significantly lifted. The suggested in this paper convergent methodology, including causal loop dynamics and eigenform realization, ensures the conditions for making the LLM inference process more purposeful and sustainable.

In the future, the transition to all-analog optical (photonic) data processing will remove the limitations created by the need to sample the signal for processing in a digital computer, which is accompanied by high energy costs and time (Raikov, 2024).

## 7 Discussion and plan for the future

### 7.1 Discussion

Generally, the main challenges of raising the explanation quality will be, in our opinion, the computational irreducibility and impossibility of creating cognitive semantics of LLM, which do not consider non-logical effects (Raikov, 2021a). Taking into account the behavior of the atomic level of the body’s and brain’s matter during the processes of human thinking requires considering their characteristics for raising the quality of XAI as follows: one neuron includes about 10^15^ atoms, neurons’ random fluctuations, 10^50^ connections between bodies and brain’s atoms, entanglement connections between the bodies and brain’s atoms and atoms of the universe.

It may be partially realized by mapping logical or neurological LLM models on images stored in holographic memory (Han et al., 2023; Raikov and Guo, 2023; Raikov, 2024). In this case, the mapping process may be accompanied by natural quantum effects: decoherence, quantum correlation (entanglement), a change in the state of quantum particles, wave function collapse, quantum non-locality, etc. Considering optical and quantum effects forces us to think about the behavior of words and other symbols in thinking and communication processes as quantum particles in the form of, for example, particles and waves or particles accompanying “shadow” particles. This allows us to raise the issue of compensating the computational irreducibility by substances of cognitive semantics. Optical and quantum interpretations of LLM models teach us that these discoveries cannot be made in a solitary and individual way: XAI has to become such a collective and resonant (or entangled) human mind, close to infinity, and only then will XAI “understand” any situation and give the relevant explanation. Experts with domain knowledge do not know everything. Still, their capabilities can be improved by relying on a cascade of other LLM systems, which can also be more capable of understanding perfectly how LLM itself reasons and produces results.

### 7.2 Plan for future

The research plan might be outlandish, but not without requirements for the above discussion because many issues and phenomena in life and science cannot be explained using traditional AI. The idea of improving the XAI explanation is based, in essence, on the local causality hypothesis proposed by David Hume. However, as shown in this article, the reason may lie beyond the limits of the foreseeable reality. Even GPT/LLM methods, ML, and cognitive modeling approaches, with their reliance on big data, are limited by using the local semantics and the retrospective field of information, digital representation of data, and so on.

Future research on creating XAI may involve considering LLM models’ poor-formalizable cognitive semantics. The critical step in advancing XAI in the future may be to consider the processing of analog signals without transforming them into digital form by creating the all-analog photonic AI (Raikov, 2024).

This draft of the plan requires interdisciplinary and international discussion and research. Unique convergent technology can make this research purposeful and stable. One variant of such convergent technology can be seen in Raikov (2021b).

## 8 Conclusion

The explanation function of GPT/LLM systems and AI as a whole is becoming increasingly crucial in further developing and applying such systems because of the need to increase confidence in the recommendations they generate.

In addition to the technological challenges, immersing the GPT/LLM system in a hybrid (human–machine) reality is decisive in improving its explainable efficiency. However, classical computer tools, such as digital linguistics, logic, autoregression, and even neuro-net tools, cannot embrace this reality. Reality requires considering non-formalizable aspects of human feeling and thinking, which digital computers cannot interpret.

It is natural to start considering algorithmic constructivism to provide new definitions of reality from the perspective of the human and the AI system simultaneously. Before providing these definitions, it is necessary to place circularity at the basis of the concept of eigenform. The eigenform is a stable object of reality created by continuous action from an AI system. It corresponds to a cognition action that requires observing in its broadest sense.

It is expected that some of the processed signals’ digital forms will need to be replaced by transforming them into analog (continuous) forms without discrete sampling. Photonic AI can bring us closer to covering the real effects of human feelings and thinking.

The growth of confidence in the inferences of XAI systems is undoubtedly facilitated by the direct inclusion of people in the process of obtaining the result. This involves achieving a continuum in hybrid reality at the interface between artificial and natural reality. For such inclusion, unique convergent technology and constructing ontologies must create conditions for making XAI, including GPT/LLM systems, more purposeful.

This article suggests convergent methodology, including all-analog photonic way, that address existing challenges and their anticipated impact on the field of XAI. In this context, elaborating on the future research directions by outlining the challenges would offer valuable guidance for advancing the domain of XAI.

## Author contributions

AR: Conceptualization, Investigation, Methodology, Supervision, Validation, Writing – original draft, Writing – review & editing. AG: Conceptualization, Supervision, Validation, Writing – review & editing. MP: Conceptualization, Funding acquisition, Investigation, Methodology, Software, Validation, Writing – original draft, Writing – review & editing. LS: Formal analysis, Investigation, Methodology, Software, Supervision, Writing – review & editing. MG: Conceptualization, Investigation, Methodology, Supervision, Writing – review & editing.

## Supplementary material

Supplementary Appendix A. Details of the stock and flow model (although there are cross-links to that in the article).
