# Supplementary material for: Accelerating human–computer interaction through convergent conditions for LLM explanation
Source: Front Artif Intell. 2024 May 30;7:1406773. doi: 10.3389/frai.2024.1406773 (PMC11177345; doi:10.3389/frai.2024.1406773)
Supplement: Supplementary Appendix A. — Details of the stock and flow model (although there are cross-links to that in the article). [file Data_Sheet_1.docx]

# Appendix A: Details of the stock and flow model

The stock and flow model presented in Figure 3 was generated and simulated with the Vensim® PLE 10.1.3 software. The following details let the reader inspect the outputs, equations, and loop definitions.

In Figure A.1-A.3, we provide the outputs of the simulations named “1st”, “2nd”, and “3rd” obtained by setting the Self-programming capability constant to 0, 0.5, and 1, respectively. A value of 0 means that the AI cannot participate in feature production; 0.5 is an intermediate and partial self-programming capability. In contrast, a value of one would allow a full-fledged capability for the AI to intervene autonomously in producing desired new features, bypassing human programmers. The output graphs are collected in groups through Figures A.1-A.4. Table A.1 shows the equations used in the model according to the Vensim syntax. Table A.2 allows us to understand and identify the causal loops classified in the model. The B loops are balancing loops, while the R is reinforcing.


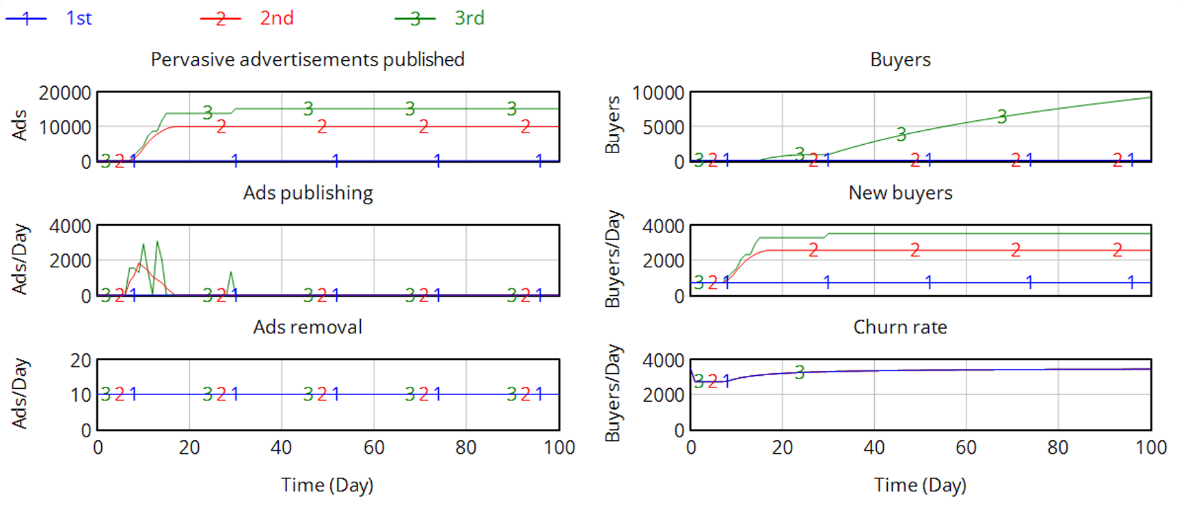


Figure A.1. First group of simulation outputs.


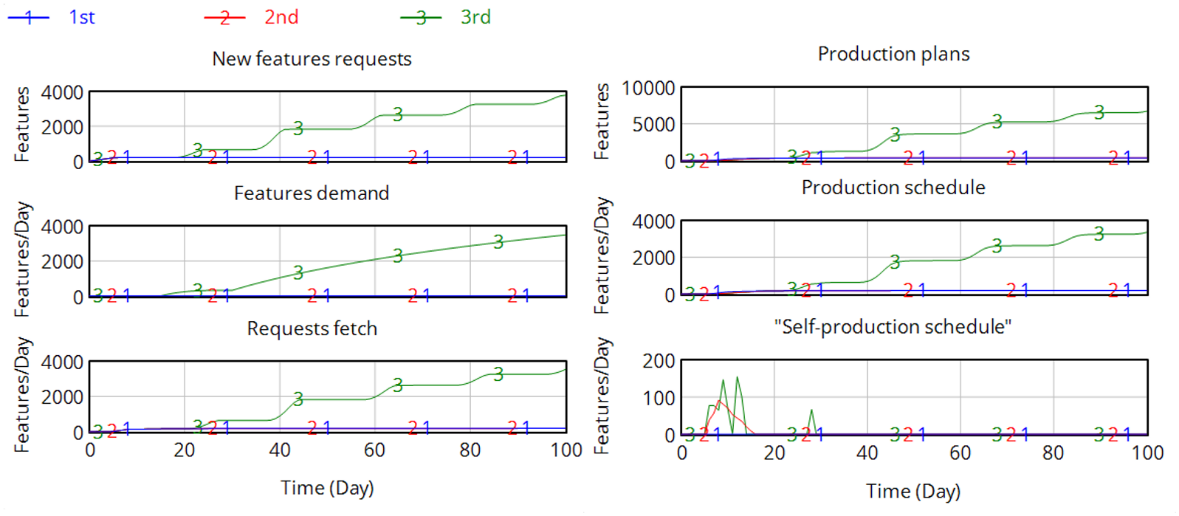


Figure A.2. Second group of simulation outputs


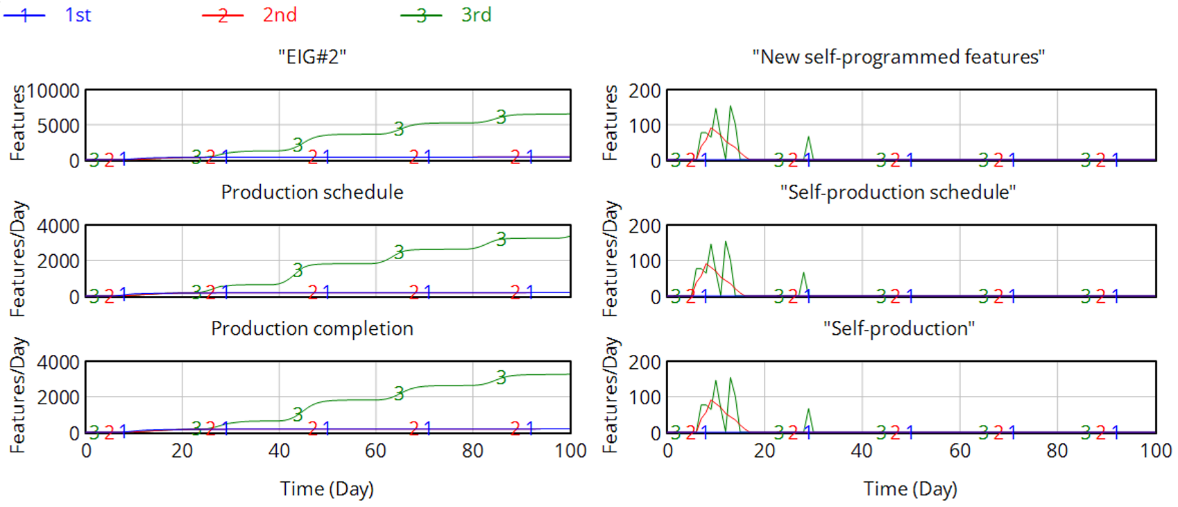


Figure A.3. Third group of simulation outputs.


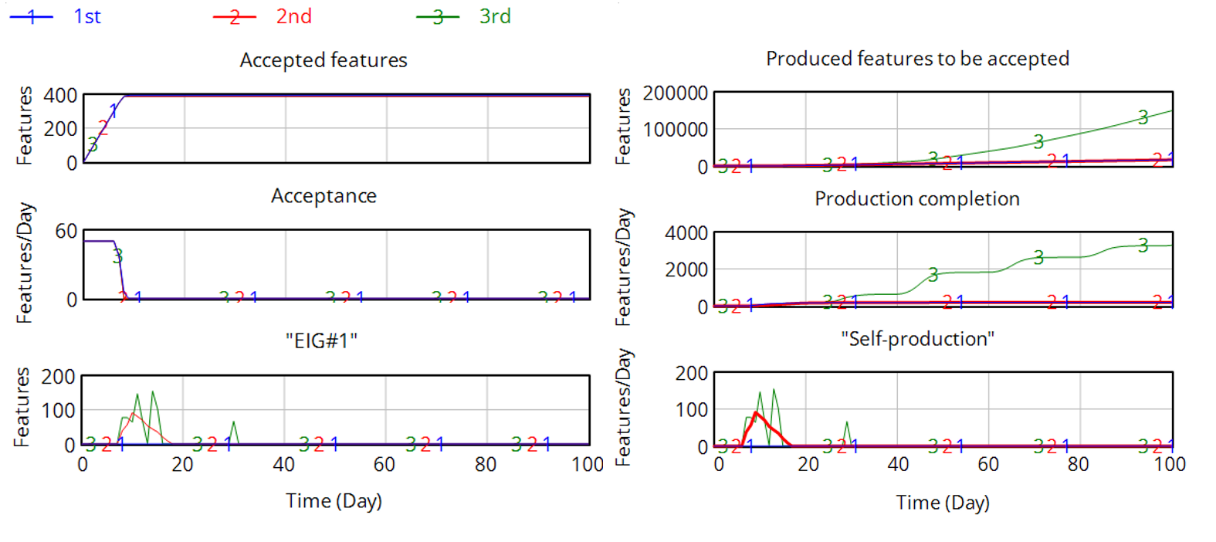


Figure A.4. Fourth group of simulation outputs.

Table A.1. Equations of the model in the Vensim format and syntax

| (01) Acceptance = MAX( (Common acceptance - ("EIG#2"+"EIG#1"))/TIME STEP, 0 ) Units: Features/Day  (02) Accepted features= INTEG (Acceptance,0) Units: Features  (03) Ads factor = 20 Units: Ads/(Features*Day)  (04) Ads publishing = Ads factor * "New self-programmed features" Units: Ads/Day  (05) Ads removal = 10 Units: Ads/Day  (06) Average new buyers per week = 5000 Units: Buyers/Day [0,10000,500]  (07) Buyers = INTEG ( MAX( New buyers-Churn rate , 0 ), 100) Units: Buyers  (08) Buyers factor = 1.3 Units: Buyers/Ads  (09) Churn factor = 0.7 Units: Dmnl  (10) Churn rate = MAX( (Average new buyers per week - ZIDZ(Accepted features* Satisfied users factor,Time)) * Churn factor , 0 ) Units: Buyers/Day  (11) Common acceptance = 50 Units: Features  (12) "EIG#1" = DELAY FIXED( "New self-programmed features" , 1 , "New self-programmed features") Units: Features  (13) "EIG#2" = INTEG ( MAX( Production schedule-Production completion , 0 ), 0) Units: Features  (14) Features demand = Features factor*Buyers Units: Features/Day  (15) Features factor = 0.379 Units: Features/(Buyers*Day)  (16) FINAL TIME = 100 Units: Day  The final time for the simulation.  (17) INITIAL TIME = 0 Units: Day The initial time for the simulation.  (18) New buyers = MAX(Average new buyers per week/7 + (Buyers factor * Pervasive advertisements published )/(7*TIME STEP),0) Units: Buyers/Day  (19) New features requests = INTEG ( MAX(Features demand-Requests fetch,0), 0) Units: Features  (20) "New self-programmed features" = INTEG ( "Self-production schedule"-"Self-production", 0) Units: Features  (21) Pervasive advertisements published = INTEG ( MAX(Ads publishing-Ads removal,0), 0) Units: Ads  (22) Produced features to be accepted = INTEG ( MAX(Production completion+"Self-production"-Acceptance,0), 0) Units: Features  (23) Production completion = MAX( ("EIG#2" - 0.5*"EIG#2")/TIME STEP , 0 ) Units: Features/Day  (24) Production plans= INTEG ( MAX( Requests fetch-Production schedule-"Self-production schedule" , 0 ), 0) Units: Features  (25) Production schedule = Production plans*0.5/TIME STEP Units: Features/Day  (26) Requests fetch = DELAY FIXED( MAX((New features requests/TIME STEP-ZIDZ(Accepted features, Time)),0) , 3 , (New features requests/TIME STEP-ZIDZ(Accepted features,Time ) ) ) Units: Features/Day  (27) Satisfied users factor = 22 Units: Buyers/Features  (28) SAVEPER = TIME STEP Units: Day [0,?] The frequency with which output is stored.  (29) "Self-production schedule" = MAX(3*Production plans - (Produced features to be accepted),0)/TIME STEP * "Self-programming capability" Units: Features/Day  (30) "Self-production" = "New self-programmed features"/TIME STEP Units: Features/Day  (31) "Self-programming capability" = 1 Units: Dmnl [0,1,0.1]  (32) TIME STEP = 1 Units: Day [0,?] The time step for the simulation. |
| --- |

Table A.2. Description of the loops in the model, by list of involved variables. Bnn are balancing loops and Rnn are reinforcing loops.

| **Loop tag** | **Entities involved in the loop** |
| --- | --- |
| **B1** | Churn rate, Buyers, Features demand, New features requests, Requests fetch, Production plans, Production schedule, IG#2, Acceptance, Satisfied users |
| **B2** | Churn rate, Buyers, Features demand, New features requests, Requests fetch, Production plans, Self-production schedule, New self-programmed features, EIG#1, Acceptance, Satisfied users |
| **B3** | Churn rate, Buyers, Features demand, New features requests, Requests fetch, Production plans, Production schedule, EIG#2, Production completion, Produced features to be accepted, Self-production schedule, New self-programmed features, EIG#1, Acceptance, Satisfied users |
| **R4** | Buyers, Features demand, New features requests, Requests fetch, Production plans, Self-production schedule, New self-programmed features, Ads publishing, Pervasive advertisements published, New buyers |
| **B5** | Buyers, Features demand, New features requests, Requests fetch, Production plans, Production schedule, EIG#2, Acceptance, Produced features to be accepted, Self-production schedule, New self-programmed features, Ads publishing, Pervasive advertisements published, New buyers |
| **B6** | Buyers, Features demand, New features requests, Requests fetch, Production plans, Production schedule, EIG#2, Production completion, Produced features to be accepted, Self-production schedule, New self-programmed features, Ads publishing, Pervasive advertisements published, New buyers |
| **B7** | New self-programmed features, Self-production |
| **R8** | New self-programmed features, Self-production, Produced features to be accepted, Self-production schedule |
| **B9** | New self-programmed features, EIG#1, Acceptance, Produced features to be accepted, Self-production schedule |
| **B10** | New self-programmed features, EIG#1, Acceptance, Satisfied users, Requests fetch, Production plans, Self-production schedule |
| **B11** | New self-programmed features, EIG#1, Acceptance, Satisfied users, Requests fetch, Production plans, Production schedule, EIG#2, Production completion, Produced features to be accepted, Self-production schedule |
| **B12** | Self-production schedule, Production plans |
| **R13** | Self-production schedule, Production plans, Production schedule, EIG#2, Production completion, Produced features to be accepted |
| **R14** | Self-production schedule, Production plans, Production schedule, EIG#2, Acceptance, Produced features to be accepted |
| **B15** | Production plans, Production schedule |
| **R16** | Production plans, Production schedule, EIG#2, Acceptance, Satisfied users, Requests fetch |
| **B17** | Requests fetch, New features requests |
| **R18** | EIG#2, Production completion |
